# The effect of a low dose hydrogen-oxygen mixture inhalation in midlife/older adults with hypertension: A randomized, placebo-controlled trial

**DOI:** 10.3389/fphar.2022.1025487

**Published:** 2022-10-07

**Authors:** Boyan Liu, Xue Jiang, Yunbo Xie, Xiubin Jia, Jiashuo Zhang, Yazhuo Xue, Shucun Qin

**Affiliations:** ^1^ Taishan Institute for Hydrogen Biomedicine, the Second Affiliated Hospital of Shandong First Medical University and Shandong Academy of Medical Sciences, Tai’an, Shandong, China; ^2^ College of Nursing, Shandong First Medical University and Shandong Academy of Medical Sciences, Tai’an, Shandong, China

**Keywords:** hypertension, hydrogen-oxygen mixture, blood pressure, randomized controlled trial, midlife/older adults

## Abstract

**Objective:** To explore the effect of a low-dose hydrogen–oxygen (H_2_-O_2_) mixture inhalation in midlife/older adults with hypertension.

**Methods:** This randomized, placebo-controlled trial included 60 participants with hypertension aged 50–70 years who were randomly divided into Air group (inhaled placebo air) or H_2_-O_2_ group [inhaled H_2_-O_2_ mixture (66% H_2_/33% O_2_)]. Participants in both groups were treated 4 h per day for 2 weeks. Four-limb blood pressure and 24-h ambulatory blood pressure were monitored before and after the intervention, and levels of plasma hormones related to hypertension were determined.

**Results:** A total of 56 patients completed the study (27 in the Air group and 29 in the H_2_-O_2_ group). The right and left arm systolic blood pressure (SBP) were significantly decreased in H_2_-O_2_ group compared with the baseline levels (151.9 ± 12.7 mmHg to 147.1 ± 12.0 mmHg, and 150.7 ± 13.3 mmHg to 145.7 ± 13.0 mmHg, respectively; all *p* < 0.05). Meanwhile, the H_2_-O_2_ intervention significantly decreased diastolic nighttime ambulatory blood pressure by 2.7 ± 6.5 mmHg (*p* < 0.05). All blood pressures were unaffected in placebo group (all *p* > 0.05). When stratified by age (aged 50–59 years *versus* aged 60–70 years), participants in the older H_2_-O_2_ group showed a larger reduction in right arm SBP compared with that in the younger group (*p* < 0.05). In addition, the angiotensin II, aldosterone, and cortisol levels as well as the aldosterone-to-renin ratio in plasma were significantly lower in H_2_-O_2_ group compared with baseline (*p* < 0.05). No significant differences were observed in the Air group before and after the intervention.

**Conclusion:** Inhalation of a low-dose H_2_-O_2_ mixture exerts a favorable effect on blood pressure, and reduces the plasma levels of hormones associated with hypertension on renin-angiotensin-aldosterone system and stress in midlife/older adults with hypertension.

## Introduction

Hypertension is a long-term medical condition that increases the risk of serious problems including heart attacks and strokes. Globally, hypertension is estimated to affect 1.4 billion people (Egan et al., 2019) and its prevalence increases with age. Among Westerners aged over 40 years, systolic blood pressure (SBP) increases by about 7 mmHg per decade (Wolf-Maier et al., 2003), and over 65% of midlife/older (aged ≥50 years) adults in the United States present with above-normal SBP (Virani et al., 2020; Craighead et al., 2021).

Lifestyle adjustments, including physical exercise, dietary changes, quitting smoking and restricting alcohol, are generally effective in lowering blood pressure. When lifestyle changes alone cannot achieve the desired result, specific medications are needed. Current drugs for hypertension include angiotensin-converting enzyme inhibitors, angiotensin II receptor blockers, calcium channel blockers, beta blockers, and renin inhibitors. The majority of patients with hypertension require a combination of at least two blood pressure lowering medications to achieve recommended goals (Bruyn et al., 2022). However, recent data have shown that less than 15.3% of individuals with hypertension in China meet the recommended target of blood pressure, and many require an increased medication dosage or additional medications to achieve blood pressure control (Bundy and He, 2016; Wang et al., 2018). In the low- and middle-income countries, only 30% of patients with hypertension had received treatment and only 10.3% had controlled hypertension (Geldsetzer et al., 2019). Thus, it is necessary to develop safe and effective adjuvant therapies to lower blood pressure.

Hydrogen (H_2_) has been regarded as a novel gaseous signaling molecule with cardiovascular therapeutic potential. A previous study indicated that 1 h of daily exposure to a low dose H_2_ (1.3%, v/v) for 4 weeks exerted an antihypertensive effect in rats with hypertension induced by 5/6 nephrectomy (Sugai et al., 2020). Meanwhile, H_2_ therapy showed benefits in alleviating hypertension-related organ damage. For instance, inhalation of 2% H_2_ gas (Matsuoka et al., 2019) or intraperitoneal injection of H_2_-rich saline (Yu and Zheng, 2012) improved left ventricular hypertrophy in different hypertensive rats, and consumption of H_2_-rich water alleviated renal injury in spontaneous hypertensive rats (Xin et al., 2014). Another clinical study demonstrated that H_2_-enriched dialysate improved blood pressure control in chronic dialysis patients (Nakayama et al., 2018). However, the effect of H_2_ in lowering hypertensive blood pressure remains controversial.

A high dose of hydrogen–oxygen (H_2_-O_2_) mixture (66% H_2_/33% O_2_) supplied *via* inhalation at a flow rate of approximately 6 L/min or 3 L/min *via* nasal cannula has been demonstrated to relieve dyspnea and other respiratory symptoms in patients with coronavirus disease 2019 (Guan et al., 2020) and to improve the quality of life in patients with cancer (Chen et al., 2019). However, the large size and expensive cost make it inconvenient for domiciliary medical care in daily life. As a simple and low-cost treatment, the clinical effects of a low-dose 66% H_2_/33% O_2_ inhalation (flow rate 30–60 ml/min) have gained increasing interest in recent years. Therefore, we performed a randomized, placebo-controlled trial in hypertensive patients aged 50–70 with baseline right arm SBP ≥120 mmHg to test the efficacy of a low-dose H_2_-O_2_ mixture inhalation in patients with hypertension.

## Materials and methods

### Study design

This study comprised a 2-week randomized, placebo-controlled, single-center, single-blind, parallel-design clinical trial that was conducted from November 2021 to January 2022. After obtaining informed consent, all participants were randomly allocated to a control Air group or H_2_-O_2_ group. The trial was approved by The Ethics Committee of Shandong First Medical University and Shandong Academy of Medical Sciences (R202107080165) and was registered at China Clinical Trial Registry (CHiCTR2100049865). All procedures were conducted in accordance with the principles of the Declaration of Helsinki.

### Participants

Potential participants were recruited from community residents aged 50–70 years diagnosed with hypertension and with established health records at Fenghuang Community Health Service Station in Tai’an, Shandong, China. Participants with right arm SBP of ≥120 mmHg by four-limb blood pressure measurement were enrolled. Individuals taking ≥3 kinds of antihypertensive drugs or with a history of major diseases and surgery, such as heart failure, malignant tumor, or organ resection, were excluded. The discontinuation criteria were withdrawal of consent, lack of compliance, or other medical reasons that required the intervention to be terminated. Participants were randomized by computer-based stratification (1:1), which was determined by age (50–59 years, 60–70 years) and gender. Allocation was concealed in opaque envelopes until the beginning of the intervention.

### Sample size calculation

An appropriate sample size (*n* = 52) was calculated using the power analysis (effect size d 0.8, α err prob 0.05, power (1-β err prob) 0.8, allocation ratio N2/N1 1) for the primary treatment outcome (G-power 3.1.9.7). To accommodate for a 15% attrition rate, we planned to recruit a total of 60 patients.

### Intervention

All participants were asked to maintain their usual diet, lifestyle, and medication. Participants in the H_2_-O_2_ group inhaled a mixture of 66% H_2_ and 33% O_2_ for a total of 4 h every day at their convenience for two consecutive weeks. The gas was generated from a H_2_-O_2_ Generator (Small H Portable Hydrogen-Oxygen Generator D-HO-001, Jinkai Instrument Co., Ltd., Dalian, China). The flow rate was approximately 30–60 ml/min, and the H_2_ concentration inhaled by the participants during breathing was about 0.2%–0.4%. The Air group inhaled normal air from a machine with the same appearance. Participants were asked to record the intervention time every day.

### Outcomes

The primary outcome in this study was the right arm SBP, and the secondary outcomes were other blood pressure variables and related blood indexes.

The values of four-limb blood pressure were automatically and simultaneously measured in an air-conditioned room at the temperature of 22–26°C *via* an arteriosclerosis detector (BP-203RPE Ⅲ, Omron, Japan) with participants in the supine position after resting for more than 5 min. A trained technician placed the pressure cuffs on both arms and both ankles for two consecutive measurements at 1-min interval, and the average value of the two results was recorded.

Assessment of the 24-h ambulatory blood pressure (ABP) monitoring was performed by an ABP detector (ABP-06, Bosheng, China). Participants were instructed to perform their habitual daily activities. The cuff was of proper size and worn on the right upper limb. ABP measurements were recorded every 30 min during the day (7:00 a.m.–10:00 p.m.) and every 60 min at night (10:00 p.m.–7:00 a.m.). The criteria for valid ABP recordings included at least 70% valid readings and at least one valid reading per hour for 21 h.

### Biochemical analysis

Blood samples after overnight fasting were collected into tubes containing EDTA at the beginning and end of the study. Plasma was isolated immediately by centrifugation at 3,000 × g for 15 min at 4°C and stored at −80°C until further analysis. Plasma creatinine, uric acid, total cholesterol, triglyceride, high-density lipoprotein (HDL) cholesterol, low-density lipoprotein (LDL) cholesterol, glucose, homocysteine, high sensitivity C-reactive protein (hsCRP), and lipoprotein associated phospholipase A2 (LP-PLA2) levels were determined using an automated chemistry analyzer (AU5821, Beckman Coulter, California, United States). Plasma cortisol, angiotensin Ⅱ, aldosterone, and renin levels were analyzed using an automatic chemiluminescence immunoanalyzer (A2000 PLUS, Antu Biological Engineering Co., Ltd, Zhengzhou, China). Superoxide dismutase (SOD) activity was measured with an assay kit (Nanjing Jiancheng, Nanjing, China).

### Statistical analysis

Data were tested for normal distribution by the Shapiro–Wilk test. Those data conforming to the normal distribution are expressed by mean ± standard deviation (mean ± SD), and the unpaired *t*-test and paired *t*-test were used to detect inter and intra group differences, respectively. Data not conforming to the normal distribution are represented by median (Quantile 1 and Quantile 3); comparisons between groups were made using Mann–Whitney *U*-test (unpaired) and Wilcoxon signed rank test (paired). Differences were considered significant when *p* < 0.05. All analyses were performed using SPSS 26.0 software (IBM, Armonk, NY).

## Results

### Participants

**FIGURE 1:**
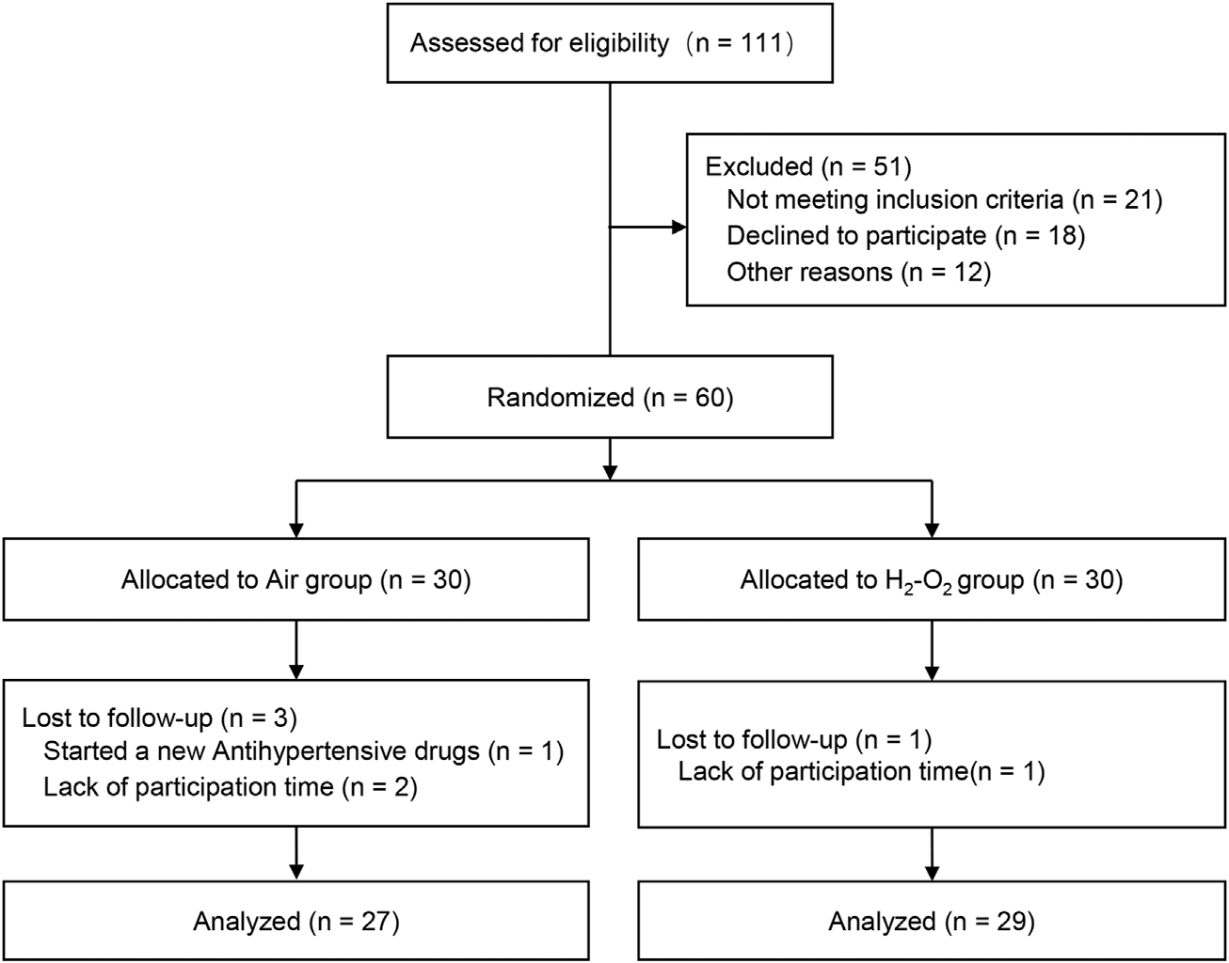
Trial flowchart.

A total of 111 participants consented to be in the study. Fifty-one participants were either excluded based on the inclusion and exclusion criteria or withdrew before study randomization. Sixty participants were finally recruited and equally randomized to the Air group (*n* = 30) or H_2_-O_2_ group (*n* = 30). During the follow-up, three participants in the Air group and one participant in the H_2_-O_2_ group withdrew from the study. Finally, 27 subjects in the Air group and 29 subjects in the H_2_-O_2_ group completed the 2-week follow-up study (Figure 1). No side effects were reported in either group. Characteristics of the participants who completed the study were well matched between the two groups, with no significant differences (*p* > 0.05, Table 1).

**TABLE 1:** Baseline characteristics of study subjects.

Characteristic	Air group (n = 27)	H_2_-O_2_ group (*n* = 29)	*P*
Female, No. (%)	16 (59.3)	12 (41.4)	0.18
Male, No. (%)	11 (40.7)	17 (58.6)	
Age	60.6 ± 6.0 (50–69)	60.6 ± 5.3 (50–69)	1.0
Weight, kg	67.5 ± 8.5	71.8 ± 8.6	0.07
Body mass index	25.7 ± 2.4	26.6 ± 3.0	0.24
Number of antihypertensive drugs, No. (%)			
0	3 (11.1)	7 (24.1)	0.20
1	18 (66.7)	16 (55.2)	0.38
2	6 (22.2)	6 (20.7)	0.89
Type of antihypertensive drugs, No. (%)			
Calcium channel blocker	13 (48.2)	9 (31.0)	0.19
Angiotensin-converting enzyme inhibitor	2 (7.4)	3 (10.3)	0.70
Diuretics	3 (11.1)	2 (6.9)	0.58
Angiotensin receptor blocker	10 (37.0)	10 (34.5)	0.84
Beta-blocker	0 (0)	2 (6.9)	0.17
Other	2 (7.4)	2 (6.9)	0.94
Concomitant diseases, No. (%)			
Currently smoking	3 (11.1)	5 (17.2)	0.51
Family history of hypertension	14 (51.9)	22 (75.9)	0.06
Diabetes mellitus	5 (18.5)	3 (10.3)	0.38
Hyperlipidemia	2 (7.0)	3 (10.3)	0.70
Coronary heart disease	3 (11.1)	4 (13.8)	0.76

Age, weight, and body mass index are presented as the mean ± SD.

Intergroup differences were tested by unpaired two-tailed *t*-tests (age, body mass index) or Pearson *χ* ^2^ test (sex, number of antihypertensive drugs, type of antihypertensive drugs, concomitant diseases).

### Blood pressure

**FIGURE 2:**
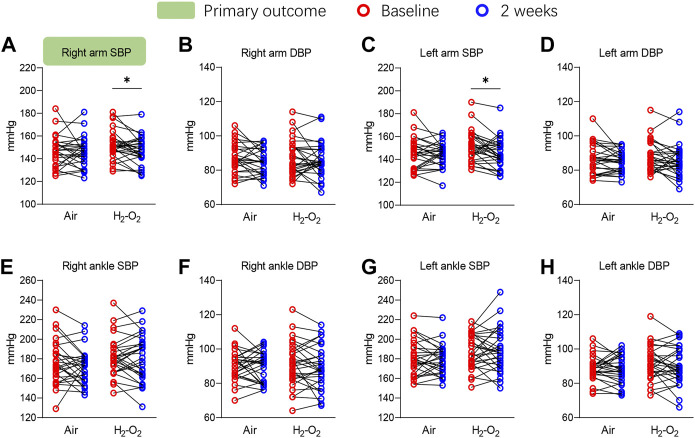
Four-limb blood pressure at baseline and after 2 weeks of intervention. SBP, systolic blood pressure; DBP, diastolic blood pressure. Data are represented as the mean ± SD; *p < 0.05 by paired *t*-test.

The primary outcome of right arm SBP significantly decreased from 151.9 ± 12.7 mmHg at baseline to 147.1 ± 12.0 mmHg after 2 weeks of H_2_-O_2_ intervention (*p* < 0.05) but was unchanged in the Air group (*p* > 0.05) (Figure 2A and Supplementary Table S1). A similar finding was noted in the left arm SBP, which showed the SBP was significantly decreased from 150.7 ± 13.3 mmHg at baseline to 145.7 ± 13.0 mmHg in the H_2_-O_2_ group (*p* < 0.05, Figure 2C and Supplementary Table S1). The diastolic blood pressures (DBP) in both arms and all the blood pressures in both ankles showed no significant treatment effect in either group (all *p* > 0.05, Figures 2B, D–H and Supplementary Table S1).

The 24-h SBP, 24-h DBP, daytime SBP, daytime DBP, and nighttime SBP did not change significantly across the intervention in the two groups (all *p* > 0.05, Table 2). A beneficial, but not significant trend, was observed for nighttime SBP in the H_2_-O_2_ group (*p* > 0.05, Table 2). Nighttime DBP decreased from 74.3 ± 9.9 mmHg at baseline to 71.6 ± 9.3 mmHg after 2 weeks of H_2_-O_2_ intervention (*p* < 0.05, Table 2) but was unchanged in the Air group (*p* > 0.05, Table 2).

**TABLE 2:** Ambulatory blood pressure.

		Air group		H_2_-O_2_ group	
		Baseline	2 weeks	Baseline	2 weeks	
24-h	SBP, mmHg	128.2 ± 8.5	129.7 ± 9.0	130.7 ± 8.1	128.6 ± 8.7
	DBP, mmHg	77.4 ± 6.7	78.5 ± 6.7	78.3 ± 9.1	77.0 ± 9.1
Daytime	SBP, mmHg	130.4 ± 8.0	132.0 ± 9.8	132.6 ± 7.9	131.2 ± 8.8
	DBP, mmHg	79.1 ± 6.5	80.4 ± 7.3	79.5 ± 9.5	78.7 ± 9.6
Nighttime	SBP, mmHg	121.0 ± 14.2	122.4 ± 13.1	124.7 ± 12.4	120.5 ± 12.6
	DBP, mmHg	72.5 ± 9.5	72.6 ± 9.2	74.3 ± 9.9	71.6 ± 9.3*

Data are mean ± SD.

*p < 0.05 vs. baseline in the same group by paired t-test.

Abbreviations: SBP, systolic blood pressure; DBP: diastolic blood pressure.

**FIGURE 3:**
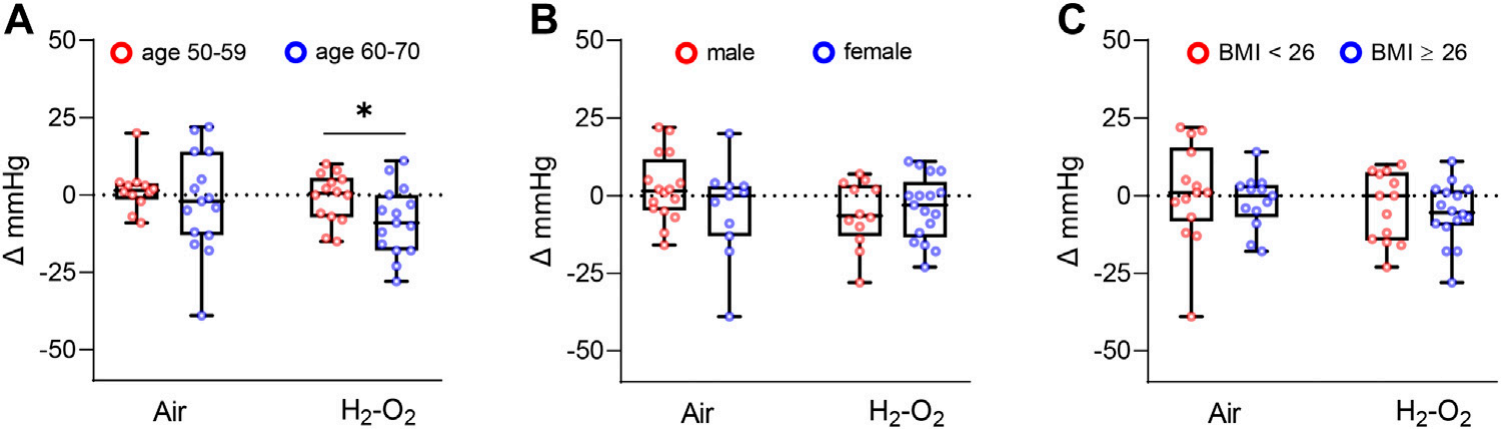
Changes in right arm SBP from baseline stratified by age**(A)**, gender **(B)**, and BMI **(C)**. BMI, Body mass index. Data are represented as the mean ± SD; *p < 0.05 by unpaired *t*-test.

Participants in the Air and H_2_-O_2_ groups were then stratified by age (aged 50–59 years *versus* aged 60–70 years), gender (male *versus* female), and body mass index (BMI) (BMI < 26 *versus* BMI ≥26). The older H_2_-O_2_ group showed a larger reduction in right arm SBP compared with that of the younger group (*p* < 0.05, Figure 3A). However, there were no significant effects according to gender and BMI (*p* > 0.05, Figures 3B,C). The reduction of right arm DBP in the older H_2_-O_2_ group was also significantly different from that of the younger group (*p* < 0.05, Supplementary Table S2). No obvious age-specific effects were observed in left arm, right ankle, and left ankle blood pressures (all *p* > 0.05, Supplementary Table S2) or ABPs (Supplementary Table S3).

### Blood assays

The renin–angiotensin–aldosterone system (RAAS) is a major hormonal cascade in the control of blood pressure. As shown in Table 3, after 2 weeks, plasma levels of cortisol, angiotensin Ⅱ, and aldosterone, as well as the aldosterone-to-renin ratio in the H_2_-O_2_ group were significantly lower compared with those at baseline (all *p* < 0.05), but there was no significant change in the Air group (all *p* > 0.05). The levels of renin showed no statistically significant changes after the either intervention (all *p* > 0.05). The H_2_-O_2_ group showed no significant changes in the plasma lipid levels of total cholesterol, triglyceride, HDL cholesterol, or LDL cholesterol; renal function indicators of urea, creatinine, or uric acid; oxidative marker of SOD activity; inflammatory indicators of hsCRP and LP-PLA2; plasma glucose; or homocysteine level (all *p* > 0.05, Table 3). We further stratified the changes in hormones related to hypertension in plasma by age (aged 50–59 years *versus* aged 60–70 years). However, these changes did not reach significant differences in each group (all *p* > 0.05, Supplementary Table S4).

**TABLE 3:** Plasma biochemical parameters at baseline and after the 2-week intervention.

	Air group		H2-O2 group	
	Baseline	2 weeks	Baseline	2 weeks
Renin, μU/ml	10.5 (6.4, 41.4)	11.6 (5.0, 31.8)	10.2 (6.8, 18.6)	9.7 (7.6, 23.2)
Angiotensin Ⅱ, pg/ml	86.3 (76.5, 117.0)	71.0 (58.0, 121.0)	92.4 (76.6, 109.5)	76.9 (57.9, 91.1)*
Aldosterone, pg/ml	165.0 (110.0, 203.0)	146.0 (113.0, 206.0)	145.0 (117.5, 192.5)	121.0 (88.4, 163.0)*
Aldosterone-to-renin ratio, pg/ml/μU/ml	10.9 (4.8, 28.1)	12.9 (4.9, 26.5)	14.5 (8.2, 19.6)	9.8 (6.3, 16.4)*
Cortisol, ug/dl	13.2 (10.1, 20.0)	13.2 (10.0, 18.1)	13.2 (11.2, 16.6)	12.9 (8.7, 15.2)*
Total cholesterol, mmol/L	5.2 (4.3, 6.0)	5.8 (4.8, 6.5)*	5.4 (4.5, 6.2)	5.4 (4.9, 6.0)
Triglyceride, mmol/L	1.7 (1.2, 2.0)	1.6 (1.2, 2.2)	1.5 (1.0, 2.1)	1.3 (1.0, 1.9)
HDL cholesterol, mmol/L	1.4 (1.1, 1.6)	1.4 (1.2, 1.6)	1.4 (1.2, 1.6)	1.3 (1.2, 1.6)
LDL cholesterol, mmol/L	3.0 (2.4, 3.6)	3.4 (2.6, 3.9)*	3.2 (2.7, 3.9)	3.1 (2.8, 3.9)
Urea, mmol/L	5.5 (5.0, 6.7)	5.9 (5.1, 7.0)	4.9 (4.4, 5.9)	5.0 (4.1, 5.8)
Creatinine, μmol/L	72.5 ± 13.9	76.2 ± 15.3*	72.9 ± 16.3	71.5 ± 13.1
Uric acid, μmol/L	293 (273, 371)	327 (286, 371)	343 (191, 399)	342 (298, 392)
SOD activity, U/ml	62.8 ± 13.9	64.3 ± 9.6	63.2 ± 9.9	65.6 ± 10.3
hsCRP, mg/L	1.5 (0.9, 2.4)	1.4 (0.8, 1.7)	2.3 (1.0, 4.1)	1.6 (1.2, 2.3)
LP-PLA2, ng/ml	109.3 (76.9,139.5)	127.7 (84.3,161.0)*	114.1 (86.7,154.4)	115.6 (88.1,151.9)
Glucose, mmol/L	6.4 (5.4, 6.9)	6.6 (5.2, 7.0)	5.9 (5.4, 7.2)	6.2 (5.5, 7.7)
Homocysteine, μmol/L	11.6 (9.4, 15.9)	12.1 (10.4, 17.5)	11.5 (9.1, 13.3)	11.2 (9.4, 13.9)

Data are median (Quantile 1, Quantile 3) or mean ± SD.

* *p* < 0.05 vs. baseline in the same group by Wilcoxon signed rank test.

Abbreviations: HDL, high density lipoprotein; LDL, low density lipoprotein; SOD, superoxide dismutase; hsCRP, high sensitivity C-reactive protein; LP-PLA2, lipoprotein associated phospholipase A2.

## Discussion

The current study conducted a randomized, controlled, parallel-design trial to evaluate the effect of a low-dose 66% H_2_/33% O_2_ mixture on the blood pressure of midlife/older adults with hypertension. We found that the 2-week H_2_-O_2_ intervention reduced right arm SBP, and the reduction in participants aged 60–70 years was significantly larger compared with that of those aged 50–59. In addition, the H_2_-O_2_ intervention improved the plasma hormone levels of cortisol, angiotensin Ⅱ, aldosterone, and reduced the aldosterone-to-renin ratio compared with the baseline levels.

H_2_ has been traditionally regarded as a biologically inert gas. However, in 2007, scholars discovered that H_2_ can exert antioxidant properties and prevent ischemia-reperfusion injury in a rat model (Ohsawa et al., 2007). Since then, a large number of studies have explored the therapeutic and preventive effects of H_2_ in different diseases, including metabolic diseases, neurological diseases, cancer, and many aging-related diseases (Liu et al., 2020; Liu et al., 2021; Fu et al., 2022).

The primary outcome of the right arm SBP was significantly reduced with approximately 4.8 mmHg after 2 weeks of H_2_-O_2_ intervention. However, the reduction in casual DBP did not reach significant difference. This may be attributable to aging is not associated with increases in DBP as reported in a previous study (Lakatta et al., 2003). For ambulatory blood pressure, H_2_-O_2_ intervention led to a decreasing trend in nighttime SBP and a significant decrease in nighttime DBP in comparison with baseline, while no changes were observed in the Air group. Certain reports have noted that sleep problems or disorders, such as sleep loss, sleep-disordered breathing, and sleep apnoea, are associated with hypertension (Nieto et al., 2000; Calhoun and Harding, 2010). A clinical study has suggested that the sleep quality of healthy people can be improved after a 4-week period of H_2_-water drinking (Tanaka et al., 2018). The influences of H_2_-O_2_ intervention on sleep and the potential effects on hypertensive are needed for further investigation.

There are several possible mechanisms by which H_2_ may lower blood pressure. The RAAS is a group of related hormones that act together to regulate blood pressure (Mentz et al., 2013). In RAAS, renin reacts with angiotensin converting enzyme to form angiotensin II, which plays a strong biological role in raising blood pressure by constricting blood vessels. It also triggers the secretion of aldosterone, which can enhance the reabsorption of sodium and water into the bloodstream from the kidney, increase the volume of fluid in the body, and also increase blood pressure (Atlas, 2007; Kobori et al., 2007). Our results show the significant effect of H_2_ inhalation on plasma angiotensin Ⅱ and aldosterone as well as the aldosterone-to-renin ratio, which may partly attribute to the blood pressure lowering effect. The detailed mechanism of H_2_ on the RAAS requires further investigation.

A higher level of the stress hormone cortisol is also linked to an increased risk of hypertension and cardiovascular events (Whitworth et al., 2000). Our research also showed that a 2-week low-dose of 66% H_2_/33% O_2_ inhalation could reduce the plasma cortisol level in patients with hypertension. It is well known that cortisol is significantly increased by psychological stressors, especially in chronic stressful events. Our result indicates that the sympathetic function of the autonomic nervous system may be suppressed by H_2_ inhalation. This calls for future studies to elucidate the mechanism of H_2_ in reducing stress.

Oxidative stress seems to be a common feature of hypertensive states. Several clinical trials have indicated that antioxidant therapy is important for the management of hypertension (Ahmad et al., 2017). H_2_ has been reported to selectively reduce reactive oxygen species (ROS) such as hydroxyl radicals and peroxynitrite anions to exert therapeutic antioxidant activity (Ohsawa et al., 2007). Previous studies demonstrated that H_2_ treatment reduced oxidative stress in different animal models with elevated blood pressure (Yu and Zheng, 2012; Guan et al., 2019). SOD is an important antioxidant enzyme that can modulate ROS levels. Studies have shown that the activity of SOD was significantly lower in the whole blood in hypertensive patients as compared to those in the normotensive subjects (Ahmad et al., 2013). However, in the present study, the oxidative marker of SOD activity did not change significantly. Many studies have demonstrated the importance of ROS production by NADPH oxidases activated by angiotensin II in hypertension (Garrido and Griendling, 2009). Thus, H_2_ may exert the free radical scavenging effects partially through angiotensin II signaling. The effect of H_2_ inhalation on oxidative stress in hypertension patient also needs further study.

Inflammatory processes play an important role in the pathophysiology of hypertension. Cytokines, toll-like receptors, and specific immune cell types are novel targets for antihypertensive therapy (De Miguel et al., 2015). A clinical trial has indicated that adding H_2_ to hemodialysis solutions can ameliorate inflammatory reactions and improve blood pressure (Nakayama et al., 2010). Wang et al. used H_2_-rich saline for the treatment of pulmonary hypertension in a rat model and reported decreased amounts of pro-inflammatory cytokines in serum (Wang et al., 2011). These results provided evidence that H_2_ improves the inflammatory processes of hypertension. However, in this study the inflammation-related biomarker hsCRP was not significantly improved, and more indicators should be tested further.

It is worth noting that the plasma levels of LDL cholesterol, creatinine, and LP-PLA2 increased in the Air group after 2 weeks, however the H_2_-O_2_ intervention showed the potentially protective effects. H_2_ has been reported to exert a wide range of therapeutic effects. Nonetheless, the underlying mechanism of its role remains ambiguous. To explore the specific molecular mechanism responsible for these effects of H_2_ will promote its clinical applications. Notably, it seems that the older the age, the more obvious the effect of the blood pressure intervention. Vascular wall thickening, elastic fiber reduction or even fracture calcification, increased peripheral resistance, decreased vascular compliance, and other factors will lead to an increase in blood pressure with age (Borzecki et al., 2006; Mceniery et al., 2007). H_2_, as a simple biomedical gas, has also been proved to be a possible antiaging agent. A recent clinical trial revealed that a 6-month intake of H_2_-rich water positively affected several molecular and phenotypic biomarkers of aging in older adults (Zanini et al., 2021). Our previous study in a D-galactose-induced aging mice model demonstrated that H_2_ administered by different routes can prevent oxidative stress and exert antiaging effects (Liu et al., 2021a). Thus, H_2_ may be used for the prevention and treatment of various aging-related diseases.

Clinically, H_2_ can be administered to humans *via* different routes, such as H_2_ gas inhalation (Tao et al., 2022), H_2_-rich water drinking (Si et al., 2021), and direct incorporation of H_2_ through diffusion (such as local hydrogen water packing (Yang et al., 2019) or H_2_-water bathing (Zhu et al., 2018)). The dose–response relation of H_2_ is worthy of deep research. A previous study compared the protective effect of H_2_ following different routes of administration in D-galactose-induced aging mice and found that although oral H_2_-water resulted in limited H_2_-intaking compared with that of H_2_ inhalation, it resulted in the same efficacy in most oxidative stress-related indicators (Liu et al., 2021). In another animal model of ischemia–reperfusion injury, lower concentrations of H_2_ (0.5%–2%) provided better effects than those of 4% H_2_ inhalation (Hayashida et al., 2008). In the present study, 30–60 ml/min of 66% H_2_/33% O_2_ inhalation showed benefits in patients with hypertension. Additional research is required to identify the optimal method and dosage of H_2_ application for different diseases.

There are several limitations of our study. First, the daily intervention times were set by the participants; thus, we were unable to identify the optimal timing. Second, although the low-dose 66% H_2_/33% O_2_ mixture played a contributing role in reducing blood pressure, the optimal concentration for H_2_ inhalation in improving hypertension deserves further investigation. Third, the sample size of the study was relatively small, and we lacked delayed follow-up data (e.g., a 6-month follow-up survey). The strengths of our study include its randomized, blinded, placebo-controlled design, the use of four-limb and 24-h blood pressure measurements, and the high compliance rate.

In conclusion, inhalation of a low-dose 66% H_2_/33% O_2_ mixture exerts a favorable effect on improving blood pressure in midlife/older adults with hypertension. According to the age stratification, the intervention was more effective among patients aged 60–70 years than among those aged 50–59 years. Possible mechanisms of H_2_ in regulating blood pressure may be the inhibitory effect on the RAAS and stress. The current findings will provide important insight into the application of H_2_ inhalation as an effective and acceptable method for patients with hypertension.

## Data Availability

The original contributions presented in the study are included in the article/Supplementary Material, further inquiries can be directed to the corresponding author.
