# Supplementary material for: The effect of a low dose hydrogen-oxygen mixture inhalation in midlife/older adults with hypertension: A randomized, placebo-controlled trial
Source: Front Pharmacol. 2022 Oct 7;13:1025487. doi: 10.3389/fphar.2022.1025487 (PMC9585236; doi:10.3389/fphar.2022.1025487)
Supplement: Supplementary file 1 [file DataSheet1.docx]

Supplementary Material

**Table S1 Four-limb blood pressure.**

|  | Air group | |  | H_2_-O_2_ group | |
| --- | --- | --- | --- | --- | --- |
|  | Baseline | 2 weeks |  | Baseline | 2 weeks |
| Right arm SBP, mmHg | 146.4 ± 13.8 | 146.0 ± 13.1 |  | 151.9 ± 12.7 | 147.1 ± 12.0* |
| Right arm DBP, mmHg | 86.7 ± 8.9 | 84.7 ± 7.9 |  | 87.4 ± 10.4 | 86.0 ± 9.9 |
| Left arm SBP, mmHg | 145.5 ± 13.3 | 144.0 ± 10.2 |  | 150.7 ± 13.3 | 145.7 ± 13.0* |
| Left arm DBP, mmHg | 86.6 ± 8.7 | 85.0 ± 6.3 |  | 87.9 ± 8.8 | 85.3 ± 9.5 |
| Right ankle SBP, mmHg | 176.3 ± 22.2 | 171.7 ± 17.6 |  | 181.2 ± 20.1 | 178.3 ± 27.6 |
| Right ankle DBP, mmHg | 90.5 ± 8.9 | 89.5 ± 8.9 |  | 91.1 ± 12.9 | 89.5 ± 12.5 |
| Left ankle SBP, mmHg | 180.6 ± 18.1 | 179.0 ± 15.2 |  | 187.6 ± 17.4 | 188.7 ± 22.0 |
| Left ankle DBP, mmHg | 88.8 ± 7.3 | 88.3 ± 8.2 |  | 91.7 ± 10.1 | 89.8 ± 10.7 |

Data are mean ±SD.* *P* < 0.05 vs baseline in the same group by paired *t*-test.

SBP, systolic blood pressure; DBP, diastolic blood pressure.

**Table S2. Changes in four-limb blood pressure from baseline**

|  | Air group | |  | H_2_-O_2_ group | |
| --- | --- | --- | --- | --- | --- |
|  | 50-59  (n = 12) | 60-70  (n = 15) |  | 50-59  (n = 14) | 60-70  (n = 15) |
| Right arm SBP, mmHg | 1.7 ± 7.1 | -2.1 ± 16.3 |  | -0.9 ± 7.9 | -8.5 ± 11.1* |
| Right arm DBP, mmHg | -0.8 ± 4.4 | -3.0 ± 9.1 |  | 2.6 ± 6.5 | -5.2 ± 10.3* |
| Left arm SBP, mmHg | 1.4 ± 7.7 | -3.9 ± 12.7 |  | -6.0 ± 12.2 | -4.0 ± 10.9 |
| Left arm DBP, mmHg | -1.0 ± 2.5 | -2.1 ± 7.7 |  | -1.6 ± 7.7 | -3.5 ± 8.1 |
| Right ankle SBP, mmHg | -1.8 ± 20 | -6.8 ± 17.4 |  | -5.2 ± 15.8 | -0.9 ± 20.1 |
| Right ankle DBP, mmHg | 0.8 ± 7.8 | -2.4 ± 10.5 |  | -0.4 ± 8.2 | -2.7 ± 9.1 |
| Left ankle SBP, mmHg | -1.0 ± 10.1 | -2.1 ± 15.3 |  | 2.9 ± 17.2 | -0.6 ± 18.9 |
| Left ankle DBP, mmHg | 0.9 ± 7.0 | -1.6 ± 6.5 |  | -1.9 ± 8.3 | -1.8 ± 8.3 |

Data are mean ± SD. * *P* < 0.05 vs 50-59 in the same group by unpaired *t*-test.

SBP, systolic blood pressure; DBP, diastolic blood pressure.

**Table S3. Changes in ambulatory blood pressure from baseline**

|  | | Air group | |  | H_2_-O_2_ group | |
| --- | --- | --- | --- | --- | --- | --- |
|  |  | 50-59  (n = 12) | 60-70  (n = 15) |  | 50-59  (n = 14) | 60-70  (n = 15) |
| 24-hour | SBP, mmHg | 2.1 ± 8 | 1.1 ± 6.3 |  | -3.2 ± 8.7 | -1 ± 6.6 |
|  | DBP, mmHg | 1.3 ± 5.7 | 0.9 ± 4.5 |  | -2.3 ± 5.7 | -0.4 ± 3.4 |
| Daytime | SBP, mmHg | 3.1 ± 7.8 | 0.5 ± 7.0 |  | -1.8 ± 8.8 | -0.6 ± 7.4 |
|  | DBP, mmHg | 2.5 ± 5.9 | 0.4 ± 5.3 |  | -1.6 ± 6.4 | -0.1 ± 4.3 |
| Nighttime | SBP, mmHg | -1.4 ± 10.4 | 3.7 ± 11.1 |  | -6.4 ± 12.7 | -2.2 ± 9.9 |
|  | DBP, mmHg | -1.9 ± 7.7 | 1.7 ± 5.3 |  | -4.2 ± 6.6 | -1.6 ± 6.4 |

Data are mean ± SD.

SBP: systolic blood pressure, DBP: diastolic blood pressure.

**Table S4 Changes in hormones related to hypertension in plasma stratified by age**

|  | Air group | |  | H_2_-O_2_ group | |
| --- | --- | --- | --- | --- | --- |
|  | 50-59  (n = 12) | 60-70  (n = 15) |  | 50-59  (n = 14) | 60-70  (n = 15) |
| Renin, μU/ml | -2.3（-33.2,1.1） | 0.7（-2.7,4.7） |  | 1.5（0.2,4.5） | 0.6（-7.5,2.1） |
| Angiotensin Ⅱ, pg/ml | -20.2（-32.2，-3.8） | -11.9（-27.3,29.7） |  | -15.3（-25.8，-8） | -22（-36.8，-0.1） |
| Aldosterone, pg/ml | -0.8 ± 58.9 | -13.7 ± 71.8 |  | -36.6 ± 44.9 | -16.9 ± 41.5 |
| Aldosterone-to-renin ratio, pg/ml/μU/ml | 0.1（-5.8,3.7） | -2.0（-9,9.3） |  | -5.9（-14.1，-1.1） | -0.7（-5.6,0.9） |
| Cortisol, ug/dl | -0.6（-2.7,1.1） | 0.8（-6.2,3.4） |  | -2.6（-7.1,0.5） | -0.3（-3.2,2.4） |

Values are median (Quantile 1, Quantile 3) or mean ± SD.
